# Medical Students’ Perceptions of Retrieval-Augmented Generation and Large Language Models: Dyadic Interview and Exploratory Survey Study

**DOI:** 10.2196/106157

**Published:** 2026-10-30

**Authors:** Rohin Athavale, Alexander Cresswell, Alice Huffman

**Affiliations:** 1 Bristol Medical School, University of Bristol, 5 Tyndall Avenue, Bristol, BS8 1UD, United Kingdom, 44 117 331 1831

**Keywords:** retrieval-augmented generation, large language models, medical education, undergraduate medical students, artificial intelligence, AI

## Abstract

**Background:**

There is evidence of rapid adoption of large language models (LLMs) in undergraduate medical education, with medical students using them to support their learning. This raises concerns about the accuracy of LLM-generated information that students are learning from. Retrieval-augmented generation (RAG), which grounds an LLM in curated materials, may be a strategy to improve the accuracy of this information.

**Objective:**

This study explored medical students’ perceptions after using an online tool, NotebookLM, that uses RAG.

**Methods:**

A cohort of 284 second-year medical students at Bristol Medical School were encouraged to use NotebookLM over a period of 4 months (March 2025 to June 2025) to help them learn course content. Following this, volunteers were recruited to share their perspectives through interviews and a survey that was open for 4 months (June 2025 to September 2025). A total of 9 individuals participated: 2 through interviews and 7 through the survey. Qualitative data were analyzed using reflexive thematic analysis and supplemented by Likert scale data.

**Results:**

NotebookLM was perceived as more reliable than standard LLM responses by interview participants, mainly due to the transparency of and control over information sources. Reliability ratings from survey participants were more mixed, but none rated unreliable. Students described it as an accessible, on-demand learning resource, which they perceived as improving efficiency and supporting self-directed study. They reported continued use of strategies to cross-check generated information. Participants demonstrated some awareness of ethical concerns, but it was unclear whether this altered their practice.

**Conclusions:**

In this small, exploratory study, students described NotebookLM as improving trust in LLM outputs while maintaining their flexibility. The findings suggest that RAG may be a strategy to support students who use LLMs. However, further research is needed to formally evaluate RAG vs standard LLMs, including the impact of RAG on accuracy, curricular alignment, and the ability of students to meet learning outcomes.

## Introduction

Medical education is notable for the volume and complexity of content that students are required to learn. This places a heavy cognitive demand on students and can make it difficult to retain and consolidate their learning effectively [1]. Large language models (LLMs) are AI models trained on extensive datasets to generate conversational “natural language” outputs based on user prompts [2]. Since the release of ChatGPT in November 2022, the popularity of LLM-based chatbots in educational settings has rapidly increased [3,4]. They offer personalized and interactive learning, yet concerns remain in areas such as their accuracy and curricular alignment [5]. Recent literature shows that LLM use is widespread among medical students, with high proportions (approximately 75%-80%) of students using LLMs to support studying and coursework [3,6]. Students with access to LLMs during medical school describe intending to use them in postgraduate training, suggesting that they perceive these tools to have high educational value [7]. However, this adoption has occurred largely in the absence of formal institutional guidance, leaving students to navigate issues of accuracy, reliability, and ethics alone [8,9]. This institutional hesitancy contrasts sharply with learner demand. Students consistently express strong interest in structured training and respond positively to LLM-supported teaching, even with limited prior experience [10]. Retrieval-augmented generation (RAG) has the potential to address a key limitation of LLMs: inaccurate or fabricated information within outputs, known as hallucinations [11]. Unlike standard LLMs, which generate responses based on patterns acquired during training, RAG-based tools retrieve information from specific external knowledge sources to anchor their outputs to verifiable information. These sources could include clinical guidelines, textbooks, or institutional teaching materials [12,13]. This is particularly valuable in medical education: grounding outputs in trusted sources may help address concerns about accuracy and curricular misalignment. In this context, RAG-based LLMs have many potential applications such as interactive learning and assessment [14]. There is currently limited evidence on how students view RAG-based LLMs in medical education [14]. Existing studies focus on technical performance, theoretical applications, and quantitative metrics such as use, with limited examination of the impact on students’ experience [15-17]. Frameworks for specific RAG-based LLMs such as NotebookLM exist, but there are few empirical studies [18].

This study explored medical students’ perspectives on using a RAG-based LLM tool, NotebookLM, in the context of medical education. It aimed to answer the following questions:

- How do students perceive a RAG-based LLM? How does it compare to other LLMs they have used previously?
- How do students feel that this RAG-based LLM tool compares to traditional study methods?
- Do students have concerns about the use of LLMs in their studies?

This exploratory study aimed to provide a basis for and direction to subsequent comparative evaluation.

## Methods

### Overview

This study primarily used a qualitative design to answer its research questions. This was to enable students’ perspectives on RAG-based chatbots to be directly and broadly explored. Some quantitative data were used to support this. This study used NotebookLM (Alphabet Inc) as a readily available, online tool based on an underlying retrieval engine. NotebookLM has features beyond a basic RAG system, for example, artifact generation and citation linking. In this study, it was used as a proxy to test students’ perceptions of RAG; however, consequently, students’ experiences of the tool cannot be attributed to RAG alone. Throughout this paper, we use RAG to refer to the technique and NotebookLM to refer to the platform.

The study adopted an interpretivist paradigm to understand the experiences of students using NotebookLM. Researchers RA and AH are lecturers in medical education at the University of Bristol. Both have an interest in AI in medical education and designed and created material for this study. This may have resulted in a more favorable reading of student perceptions during analysis and placed more emphasis on educator-facing concerns rather than student-facing concerns. AC is a medical student from the cohort below those recruited to the study and has an interest in AI in health care. Researcher AC provided a valuable viewpoint during analysis, helping develop candidate themes from a student perspective and ground reported student perceptions.

This study was conducted at the University of Bristol using second-year medical students. Second-year students were chosen because they were likely settled into learning in higher education and may be experimenting with LLMs to assist with their studies.

This cohort of 284 students was encouraged to use NotebookLM over a period of 4 months (March 2025 to June 2025) through Gemini Notebook (free tier) [19]. NotebookLM’s underlying model was Gemini 2.0 Flash (Alphabet Inc; before May) and Gemini 2.5 Flash (Alphabet Inc; after May). Students were directed to upload and use an educator-created corpus of 6 protocols on clinical examinations, outlining how to perform clinical examinations, through the NotebookLM website. Training was provided, including written guidance on how to set up NotebookLM with learning materials and suggestions on how to best make use of the tool (Multimedia Appendix 1), along with contact details of researchers to contact if students required additional support. Given that NotebookLM is a proprietary Google product accessed through students’ personal accounts, researchers did not have direct access to student use data for the tool. Beyond year of study and self-reported AI use, no participant characteristic data were collected to prevent potential identification.

Following this period, the whole cohort of 284 students was invited to volunteer to join a focus group or complete a survey. Thirteen students expressed interest in joining a focus group; however, ultimately, only 2 met with researchers, resulting in the focus group being run as a dyadic interview (June 2025). The survey was open for 4 months (June 2025 to September 2025); 7 students completed the survey (Multimedia Appendix 2). There was no overlap in participants between the 2 methods. Students were recruited via messaging in their weekly student bulletin, distributed to all second-year students via email, with an invitation to participate advertised throughout the survey period.

The dyadic interview was conducted jointly by 2 researchers (RA and AH). The interview lasted approximately 1 hour and was guided by a topic guide (Multimedia Appendix 3). It was conducted on Microsoft Teams, which was used to record and transcribe the meeting. The transcription was manually cleaned and anonymized prior to analysis. The survey was completed by participants in their own time using Microsoft Forms.

Qualitative data collected consisted of the dyadic interview transcript and free-text survey questions. These were analyzed using reflexive thematic analysis following the approach set out by Braun and Clarke [20,21], treating data as self-reported perceptions rather than necessarily complete reports of practice, and using inductive analysis to build themes.

Researchers RA and AH familiarized themselves with the complete anonymized datasets and independently co-coded the data before peer debriefing to discuss candidate themes. Discussion was collaborative, rather than focused on reliability, and divergent analysis was used to refine and enrich themes. Interview and survey responses were initially coded as distinct sources, with interview data analyzed first. These codes were then combined as themes developed, with the sources of quotes noted. Three levels of codes were formulated before being built into themes: (1) simple codes describing participant quotes; (2) basic grouping of ideas; and (3) further grouping, focusing on meaning. Final themes were then discussed, grounded in extracted codes and quotes, and reviewed by all researchers. Divergent opinions were specifically attended to, used to temper themes, and are reported in the results where applicable. Supporting quantitative data consisted of Likert scales, which were visualized and analyzed descriptively.

Given the use of reflexive thematic analysis, researchers did not focus on concepts such as accuracy or consensus as quality markers. Credibility was supported by 2 researchers analyzing the whole dataset, with differing readings adding value to themes, and a third researcher, who occupied a position closer to participants, also contributing to candidate themes. Dependability was addressed by describing the analytical sequence in the preceding paragraph and making the instruments available in the multimedia appendices. Researchers’ analyses were recorded in shared documents and Microsoft Excel sheets. Confirmability was supported by providing labeled extracts as evidence for each theme, allowing readers to assess their fit. Researchers’ positioning and influence on analysis are noted above. Transferability was addressed by describing the setting, cohort, and intervention. The findings derive from 9 students in a single UK medical school. Researchers do not suggest that the results are transferable beyond comparable settings.

The dyadic interview preceded the survey, with 2 participants, and there were 7 respondents to the survey, resulting in a total of 9 (3.2% of the cohort) participants. Given the focused aim, this study was guided by the concept of information power [22]. Two domains best support this: our study aim was narrow, focusing on a single tool and context, and the sample was specific, comprising a relatively homogeneous group of students. The dyadic interview provided a richer quality of dialogue and bolstered data from the survey. This study is weaker in the domains of analysis strategy and use of established theory. Although breadth was very limited, the dataset demonstrated enough depth to provide some insights into students’ perspectives on RAG. Nevertheless, the analysis should be read as an early, partial account of student experiences.

### Ethical Considerations

Ethics approval for this study was granted by the University of Bristol’s Faculty of Health Sciences Ethics Committee (23448). Participation in the study was voluntary. All participants were provided with participant information sheets and contact information for further questions, and all participants completed a written form supplying informed consent before data collection via Microsoft Forms.

Participants were able to withdraw from the study at any point up until data anonymization. Data from the dyadic interview and survey were anonymized prior to analysis. Both Microsoft products were accessed using only Bristol University–affiliated accounts, and all data were stored on secure Bristol University devices.

Participants could optionally enter a prize draw for Love2shop vouchers, with the chance to win a £50 (£1=US $1.34, as of October 21, 2026) voucher for the dyadic interview and £20 for the survey, as outlined in the participant information sheet.

Researchers RA and AH regularly taught the cohort and contributed to question writing and standard setting for the multiple-choice question (MCQ)–based examination for this cohort. Both made clear that this research project had no bearing on students’ normal education. Additionally, students were recruited via the weekly bulletin rather than in teaching sessions; the advertisement and participant information sheets highlighted that participation was voluntary and that data would be anonymized prior to analysis. Given the researchers’ positions, there was a risk of favorable reporting. To compensate for this, the topic guide was written with neutral framing throughout and included questions explicitly asking for negatives.

A university data protection impact assessment (DPIA) was completed for the use of NotebookLM in this study. This included a review by researchers and university information governance staff of NotebookLM’s data processing. Training included a data privacy statement outlining anticipated data use and instructions not to include identifiable or sensitive information. Students were instructed to upload 6 clinical examination protocols authored by the researchers ahead of this study. Students were advised not to upload copyrighted content, such as lecture slides, without the consent of the copyright holder. However, given that researchers had no control over how students interacted with this tool, students could upload other materials and use them with NotebookLM.

## Results

### Participant Information

Results capture 9 separate students (2 in the dyadic interview; 7 in the survey). All participants were second-year medical students: quotes are attributed to interview participants as DI-1 and DI-2 and to survey respondents as S-1 through S-7. Use of NotebookLM was varied, and 1 participant (S-6) reported never using LLMs or NotebookLM (Table 1). Interview participants described active use of the tool, resulting in 6 of 9 participants having meaningful exposure to the tool.

**Table 1.** Self-reported AI use among survey respondents (S-1 to S-7), including previous use of AI tools for educational purposes and use of NotebookLM during the study period^a^.

Respondents	Frequency of use of AI tools other than NotebookLM	Frequency of use of NotebookLM during the study period
S-1	Multiple times a week	Multiple times a week
S-2	Multiple times a week	Rarely
S-3	Daily	Daily
S-4	Multiple times a week	A few times a month
S-5	A few times a month	Multiple times a week
S-6	Never	Never
S-7	A few times a month	Rarely

a Three respondents (S-2, S-6, and S-7) reported minimal exposure to NotebookLM. The data reflect these students’ perceptions of NotebookLM and, where applicable, their retrospective views of other large language models. Full Likert scale survey data are available in Table 2, and the thematic structure is presented in Table 3.

**Table 2.** Complete Likert scale and categorical survey data (N=7)^a^.

Survey questions and Likert descriptors	Participants, n
Excluding NotebookLM, how often do you use AI tools (eg, ChatGPT) in your studies?	
None	1
Rarely	0
A few times a month	2
Multiple times a week	3
Daily	1
Excluding NotebookLM, how useful do you find AI tools for your learning?	
Not useful at all	1
Somewhat not useful	0
Neutral	0
Somewhat useful	3
Very useful	3
How frequently did you use NotebookLM while it was available	
Never	1
Rarely	2
A few times a month	1
Multiple times a week	2
Daily	1
How *useful* did you find NotebookLM?	
Not useful at all	0
Somewhat not useful	0
Neutral	3
Somewhat useful	3
Very useful	1
How *reliable* did you find the information provided by NotebookLM?	
Very unreliable	0
Somewhat unreliable	0
Neutral	3
Somewhat reliable	3
Very reliable	1
How does using NotebookLM compare to traditional study methods in terms of *effectiveness*?	
Much less effective	0
Somewhat less effective	1
About the same	2
Somewhat more effective	2
Much more effective	2
How does using NotebookLM compare to traditional study methods in terms of *efficiency*?	
Much less efficient	0
Somewhat less efficient	1
About the same	2
Somewhat more efficient	2
Much more efficient	2
How concerned are you about your data when using AI tools such as NotebookLM for your studies?	
Very unconcerned	1
Somewhat unconcerned	0
Neutral	0
Somewhat concerned	4
Very concerned	2
How concerned are you about the environmental impact of using AI tools such as NotebookLM for your studies?	
Very unconcerned	1
Somewhat unconcerned	0
Neutral	1
Somewhat concerned	1
Very concerned	4
If you had access to a tool such as NotebookLM, would you favor it over traditional study methods?	
No	2
Unsure	3
Yes	2
Would you recommend using NotebookLM to other medical students?	
No	2
Maybe	0
Yes	5

a Given the small sample size, results are reported as counts and are not used to strengthen claims beyond the qualitative evidence.

**Table 3.** Thematic structure outlining the final themes, their central concepts, and the participants contributing qualitative evidence from the dyadic interview (DI; participants DI-1 and DI-2) or the survey (S; participants S-1 to S-7).

Themes	Central concept	Contributing participants (total number of participants)
Pragmatically reliable	Reliability is often judged as “good enough” and in comparison with other knowledge sources.	DI-1, DI-2, S-4, and S-7 (4)
Flexible study catalysts	Students perceive LLMs^a^ as accelerating existing workflows rather than superseding them.	DI-1, DI-2, S-4, and S-7 (4)
Ethical awareness but limited action	Students have ethical concerns related to LLMs, but it is unclear whether these concerns alter their practice.	DI-1, S-5, S-6, and S-7 (4)
Expansion outpacing guidance	Some students adopt LLMs ahead of formal approval or training. Some are optimistic about future education opportunities provided by LLMs.	DI-1 and DI-2 (2)

a LLM: large language model.

### Pragmatically Reliable

Students were simultaneously aware that LLMs can make mistakes but still broadly rated them as reliable. They compared hallucinations with inaccuracies found in textbooks or when trying to find an answer through Google Search (Alphabet Inc). These seem to be the benchmarks against which some students rate LLM reliability:

I think it generally for my purposes it’s more accurate than it isn’t.

[DI-1]

I think it’s a good source compared to possibly going back to a textbook we were [using] before.

[DI-2]

Students expressed the need to be critical of LLM outputs, and some reported techniques to corroborate information generated by LLMs against other information sources:

You need to be very certain that what are whatever you’ve been kind of relayed back to is correct as well, so it’s not like blindly following it.

[DI-2]

You don’t just use one tool. You’d cross reference for example with your textbook with your lecture slides.

[DI-1]

Students found that NotebookLM increased their confidence in model outputs. The ability to provide trusted sources increased the tool’s transparency and students’ sense of control over the tool. Students reported that NotebookLM also allowed customization to suit the student’s institution, for example, by aligning with intended learning outcomes (ILOs):

Yeah, I mean, I suppose it does kind of solve that in, in a sense solve that problem of hallucinations.

[DI-1]

Before revising the lecture, I open up a document word, and then I put the learning outcome and then basically have an actual explanation of each of the learning outcome... now, as you can see with the notebook[LM], it provides you a very good broad. Like kind of a summary of the document itself.

[DI-2]

On the other hand, 1 student had concerns about accuracy. Another expressed apprehension about using a new tool but ultimately found it useful:

I am more concerned that the information is unreliable.

[S-4]

It was a new AI tool so I was slightly reluctant to use but it was a nice tool to generate new questions.

[S-7]

Mixed perceptions of reliability were reported in Likert scale data, with 1 of 7 students rating NotebookLM as very reliable and 3 rating it as somewhat reliable. On the other hand, 3 rated reliability as neutral, which for some may reflect limited exposure, given that 2 of these students used NotebookLM “never” or “rarely.”

### Flexible Study Catalysts

Students viewed NotebookLM as an on-demand study partner. They reported that it was easy to use, accessible, and increased their perceived productivity. Students used the tool to summarize information, answer questions, and generate study materials. Participants described it as accelerating and complementing existing practices, rather than completely replacing them:

It’s on demand. It’s accessible, it’s online. I don’t need to necessarily be in the classroom to do it.

[DI-1]

I found NotebookLM useful in generating multiple choice questions or written answer questions to test me on examination findings.

[S-4]

Students felt that the tool increased their productivity and efficiency. Of the 7 survey respondents, 4 found the tool useful, while 1 reported finding NotebookLM less efficient and effective:

You can free up time and you can do more lectures.

[DI-2]

I find AI useful in creating multiple choice questions and testing myself on revision. I use it to identify areas of struggle that I can then research in the course content.

[S-4]

There were several instances in which students compared NotebookLM to lecturers. Some stated that they found the tool more accessible than lecturers, as it can instantly answer questions and they felt less pressure than when asking a lecturer:

We don’t really have office hours in medicine... I would think of it as like a on demand office hour.

[DI-1]

The language model will give you more time to think, or potentially even say “that’s not a very good question.” Like “is there a better way to word that?”

[DI-1]

I felt AI was useful for medicine learning to simplify complex topics and its accessibility to ask questions rather than having to chase and ask lecturers.

[S-7]

There was no qualitative evidence that students were using LLMs to completely replace other information sources. This is consistent with the mixed response to the survey item “would you favor it over traditional study methods”: 2 responded no, 3 were unsure, and 2 responded yes.

### Ethical Awareness but Limited Action

Participants demonstrated some awareness of ethical issues related to LLM use in education. However, for many students, there was limited evidence of whether or how this changed how they used LLMs. On the other hand, for 1 participant (S-6), their reported abstention from using LLMs could be linked to their perceived ethical concerns.

A major concern was the data implications of using LLMs—most survey respondents (6/7) expressed concern regarding this. Students reported awareness of other risks of using LLMs, for example, environmental impact and students becoming overly reliant on AI:

You’re giving your your data or your piece of work to the company who runs the AI... and then now they have this information [that you] don’t really know what they do with it, regardless of what data laws we have...

[DI-1]

Concern: over reliance on AI education may reduce the value of education from clinical experiences.

[S-7]

Additionally, 2 students identified bias as a potential issue within LLMs, for example, the potential for misdiagnosis of patients belonging to minority groups:

I’m worried about AI bias especially with regards to how they may see and misdiagnose POC and female patients which may further contribute to ongoing bias. I also worry that as work becomes more specialised that AI is more likely to be incorrect.

[S-5]

AI having the same biases of its inputs leading to similar racial biases that already exist in healthcare being exacerbated.

[S-6]

### Expansion Outpacing Guidance

This theme is derived predominantly from dyadic interview participants, with some Likert responses used for context.

Some students already use AI and LLMs such as ChatGPT (OpenAI) for tasks in their personal lives and in their studies. Common uses include concept clarification and summarization. Some have independently experimented with and incorporated these tools into their digital learning repertoire, despite minimal guidance or support from teaching institutions:

I’m looking at my current like history on ChatGPT and it’s basically all like work related.

[DI-1]

So that if the teachers or the lecturers don’t accept it, the students are still going to use it anyway.

[DI-2]

This is consistent with survey data showing that the majority (6/7) of respondents reported that they used AI tools such as ChatGPT at least a few times a month in their studies. The majority (6/7) also reported that they generally found AI tools somewhat or very useful for their learning. The remaining respondent reported never using AI tools and finding them “not useful at all.”

Looking forward, students in the dyadic interview reported optimism about the use of AI in medical education. They envisioned LLM-based tools providing additional educational opportunities and helping to personalize learning. Students also perceived value in the use of LLMs in other domains, for example, practicing for objective structured clinical examinations (OSCEs):

I would definitely see in the future that AI would play a bigger role because that’s just a natural progression of it.

[DI-1]

There’s still a lot of lot of potential for it to go everywhere and be everywhere... regardless of the downsides.

[DI-1]

If I wanted to practise further at home. Particularly before... an OSCE exam when you don’t have anybody to practise with, then yes.

[DI-2]

They reported that RAG was a powerful addition to LLMs that could help facilitate this. Of the 7 survey respondents, 5 said they’d recommend NotebookLM to other medical students, while 2 would not.

## Discussion

This study explored medical students’ perspectives on a RAG-based LLM tool, NotebookLM, in an educational context. This included students’ perceptions of the reliability of RAG-based LLM outputs, how they compared these outputs with those from traditional study methods, and ethical concerns they had about using such systems.

Findings suggest that, for some students, LLMs have become embedded in their learning workflows. Reported data suggest that, rather than replacing traditional study methods such as lectures or textbooks, LLMs may be used as an additional tool within students’ digital repertoires. Therefore, some students appear to be shaping their learning environment into one in which traditional and AI tools coexist, rather than relying on LLMs alone [23].

Consistent with previous research, students reported that a key benefit of using LLMs was increased perceived efficiency, with students describing feeling able to cover more content and save time [5,24]. They appear to value the flexibility to use them as part of their self-directed learning in a manner similar to an on-demand tutor. It was interesting to see LLMs being directly compared to lecturers, with LLMs described as more accessible and less intimidating than interacting with staff. Given increasing cohort sizes, it is perhaps logical that learners would seek bespoke, individualized support through LLMs [17].

Students were aware that model outputs could be inaccurate and reported strategies to evaluate the outputs used during their studies. They described actively verifying information against other sources, such as textbooks, suggesting ongoing critical appraisal of LLM outputs. Such behavior challenges the idea that LLMs always lead to shallow learning and uncritical reliance [25]. Perceptions in this study suggest that some students find LLMs reliable enough to be useful and are aware that other sources they may use, such as textbooks, are also imperfect.

Interview participants were positive about the incorporation of RAG into LLMs. Participants described how controlling sources improved their confidence in the LLM’s output. The drivers of this increased trust were the transparency of sources, the ability to define the supplementary knowledge base, and the ability to align that knowledge base with the learners’ own needs. In particular, the ability to align a model with institution-specific ILOs and content was valued.

From the perspective of educators, the incorporation of RAG likely provides some reassurance about the standard of content that students are interacting with. The ability of educators to view and interrogate underlying sources is likely of educational benefit. Additionally, educators could provide sources to be used in a RAG-based LLM to guide students’ learning and improve their confidence that students are learning accurate information. However, formal evaluation of these possibilities is required.

Although students were generally positive, many had ethical concerns about data handling, bias, overreliance, and the environment. This may form a barrier to some students engaging with LLMs in medical education. It is important to note that these concerns may contribute to why 1 student reported never using LLMs. Different use patterns may risk widening the digital gap between students who do and do not use AI [26].

Students were often unable to articulate these risks fully. It was also unclear if and how this awareness changed their practice and how they interacted with LLMs. For example, they broadly did not discuss strategies to identify or mitigate bias in outputs. Taken together, this suggests that medical students may benefit from formal education on AI, including ethical considerations, such as on how the environmental impact of LLMs compares with that of other digital activities [27,28].

These findings may point to a changing relationship between educational bodies and LLMs. The increasing use of LLMs among medical students suggests that institutions may need to take a more active role in guiding and supporting students using AI tools [29]. RAG-based LLMs may be an immediate and relatively easy way to support students who choose to use LLMs in their studies and improve perceived output accuracy, although further research in this area is required, especially to evaluate the actual accuracy of RAG-generated outputs.

The major limitation of this study is the small number of respondents (3.2% of the cohort). This study required independent use of NotebookLM and engagement with researchers, meaning that the study likely captured students who were enthusiastic about using LLMs. This likely means there is unexplored breadth and depth in the themes identified in this study. Students also had access to the tool for only 4 months, and objective data on how frequently students used NotebookLM and for what purposes were unavailable. Some survey respondents reported low use of NotebookLM. Results were based on the perceptions of this small subset and may not reflect the wider cohort’s views; participants may be more positive than other medical students. Additionally, NotebookLM was used as a proxy in this study, and perceptions of NotebookLM may not translate to other RAG-based tools.

Further research in this area could include more students to further explore the themes identified in this study. This could also identify whether there are differences in medical students’ perspectives of RAG across year groups, according to variable prior experience with AI, or between institutions. Furthermore, it would be valuable to investigate whether using RAG-based LLMs impacts other measures of learning, such as achievement of ILOs or academic performance.

Overall, this exploratory study suggests that some students perceive a RAG-based LLM as beneficial to their learning compared to standard LLMs, enhancing their perceived reliability and trust in LLM outputs while supporting flexible learning. For some, LLMs have become embedded in their learning practices, despite a perhaps underdeveloped understanding of the ethical implications of AI use. These findings suggest a route by which medical educators and institutions can guide the integration of AI into education. Further research formally comparing RAG-based LLMs to other information sources would be valuable, especially to investigate the impact on educational outcomes. Additionally, this should be accompanied by broader efforts to develop AI literacy and critical engagement with these tools.

## Supplementary material

10.2196/106157 Multimedia Appendix 1 Training material provided to students to support their use of NotebookLM.

10.2196/106157 Multimedia Appendix 2 Full survey including question items and answer choices.

10.2196/106157 Multimedia Appendix 3 Topic guide used during dyadic interview.

## Abbreviations

DPIA: data protection impact assessment
ILO: intended learning outcome
LLM: large language model
MCQ: multiple-choice question
OSCE: objective structured clinical examination
RAG: retrieval-augmented generation
