# Supplementary material for: Medical Students’ Perceptions of Retrieval-Augmented Generation and Large Language Models: Dyadic Interview and Exploratory Survey Study
Source: JMIR Form Res. 2026 Oct 30;10:e106157. doi: 10.2196/106157 (PMC13722098; doi:10.2196/106157)
Supplement: Supplementary file 1 [file formative-v10-e106157-s001.pdf]

Introduction

Thank you for your interest in this research study on how large-language models could benefit students in medical education. As part of this study, we're signposting to and offering training on how to use NotebookLM to learn clinical examinations to all second year medical students.

A large-language model (LLM) is an AI tool that allows you to 'talk' to the model based on it's knowledge base, for example ChatGPT. A LLMs accuracy can be improved by providing it documents to reference, and restricting it's answers to these documents, a process known as retrieval-augmented generation. We have chosen NotebookLM as it is a Google LLM that uses retrieval-augmented generation.

This document will outline how to get started using NotebookLM, example uses, and a FAQ.

Note: as with any LLM, we strongly recommend you avoid inputting any personal data (name, date of birth, address etc.) of yourself or others.

NotebookLM is free to use with a Google account.

https://notebooklm.google.com/

Data privacy

NotebookLM is a Google product.

If you use NotebookLM with your personal Google account, Google may link data collected by NotebookLM to your account. This may data such as text entered into NotebookLM and your IP address. We suggest creating a new Google account if you'd like to try NotebookLM without potentially linking data to your personal account.

We don't anticipate NotebookLM directly collects identifiable or sensitive data. **Please do not input identifiable or sensitive data** into the text field when using NotebookLM. You can read more about how NotebookLM may use your data in Google's privacy policy: https://policies.google.com/privacy?sjid=14290412534519072733-EU

Google have stated that model input and responses are not used to train future models: https://support.google.com/notebooklm/answer/15724963?hl=en

Getting started

1) Navigate to https://notebooklm.google.com/ and sign in using, or create a Google account

If you get a 'service unavailable' message, you may need to verify your age on your Google account

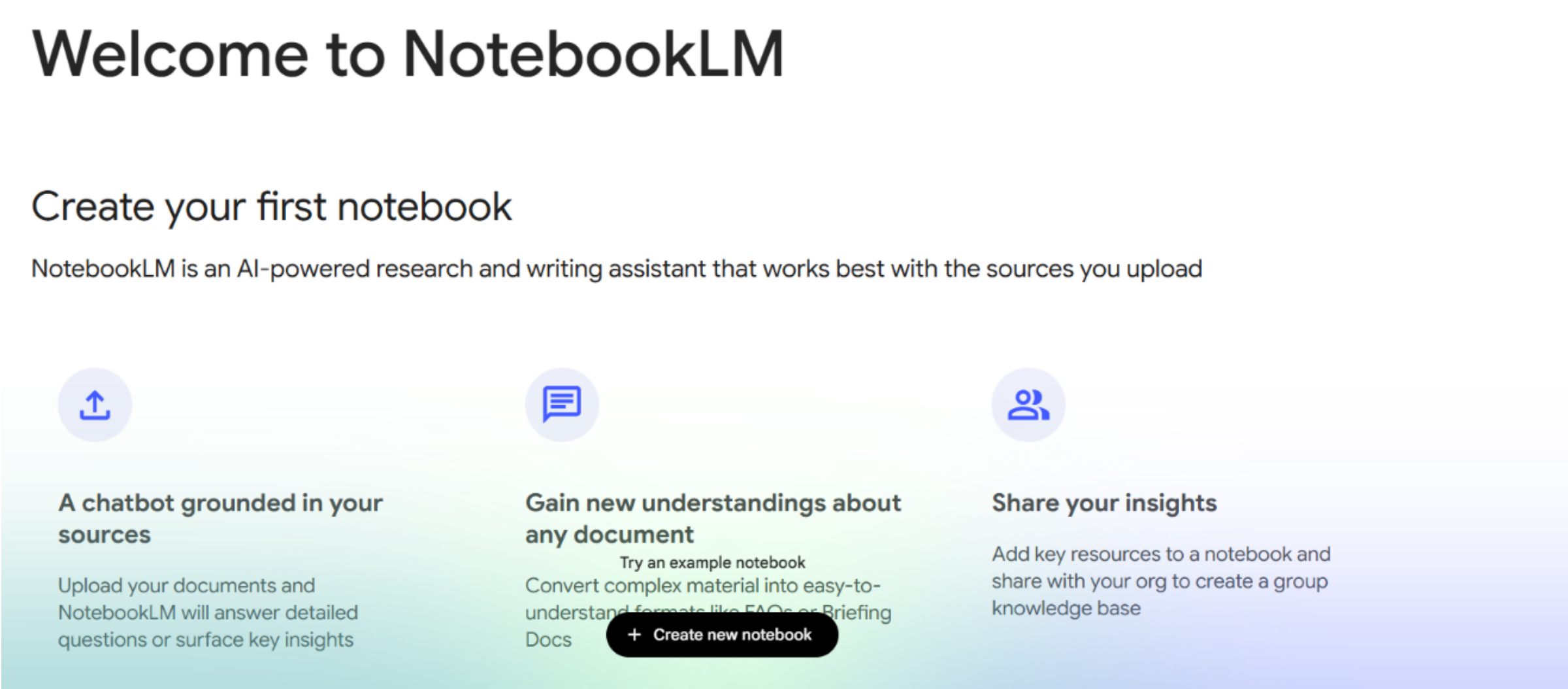

2) Click "+ Create new notebook" at the bottom of the screen

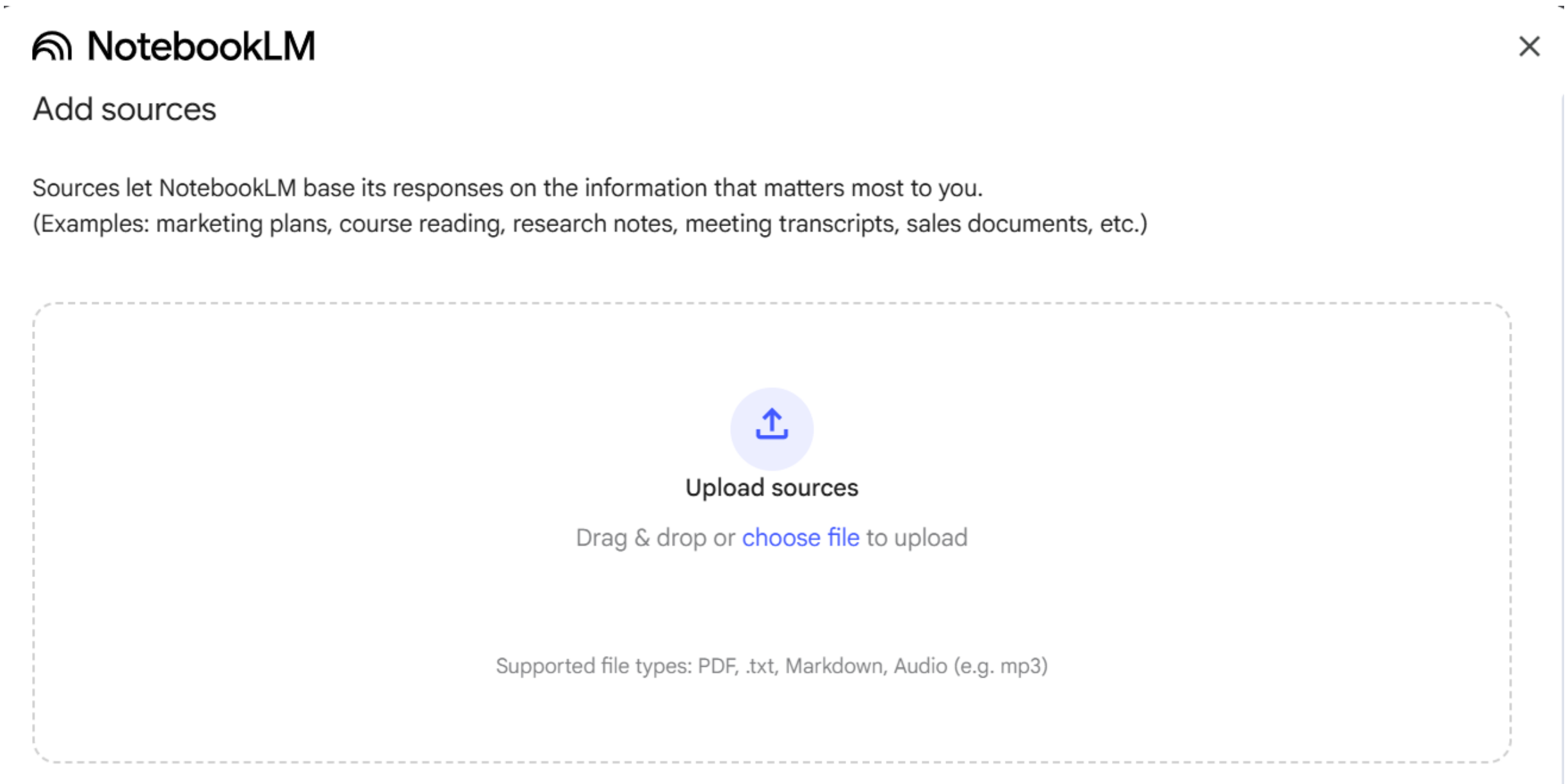

3) You'll be prompted to 'upload sources'. Upload the 6 examination protocol pdfs and the 'MCQ style guide' file. These are available in the 'examinations for upload folder'

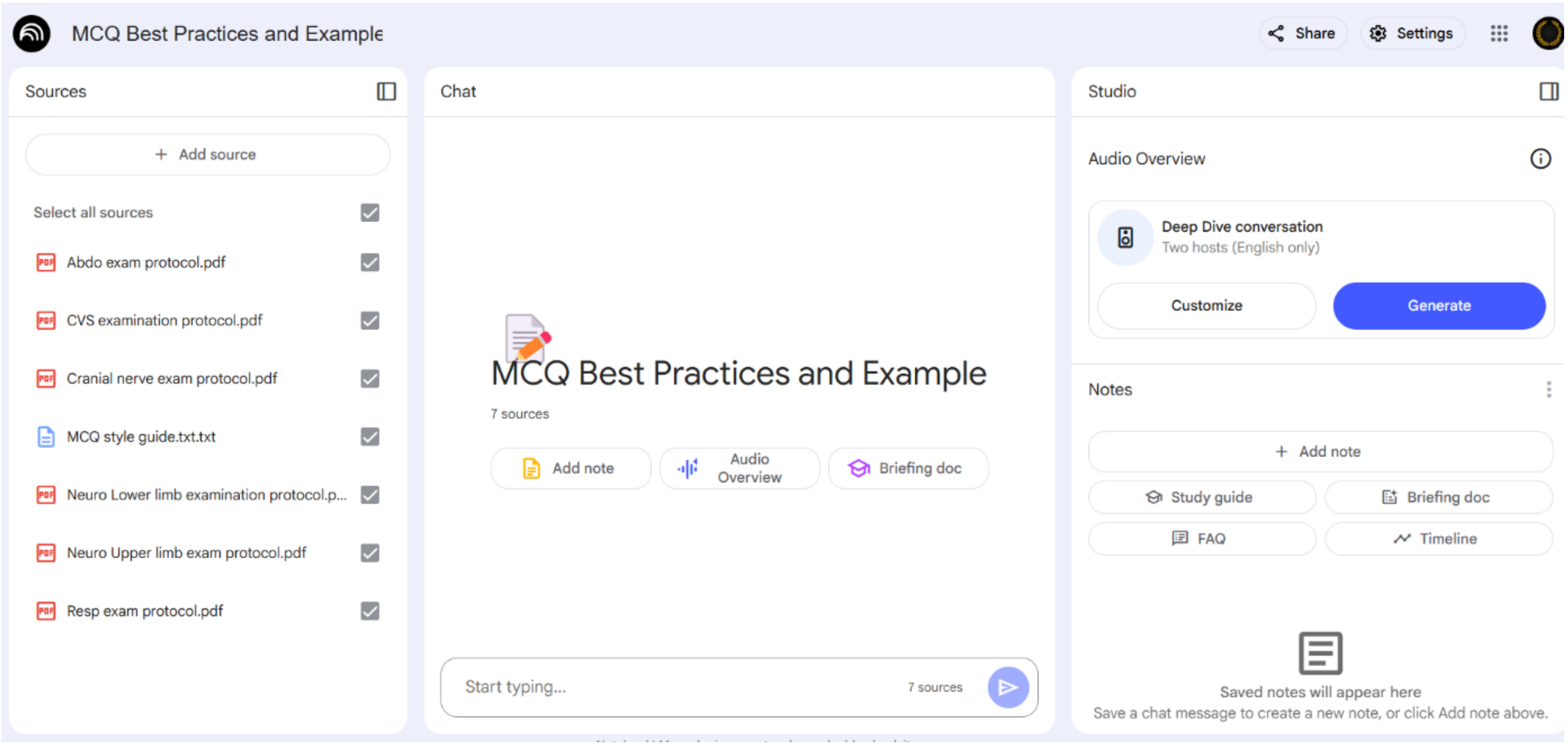

4) You should now see a new notebook with the uploaded files displayed on the left hand side. Try asking a question about clinical examination in the chat.

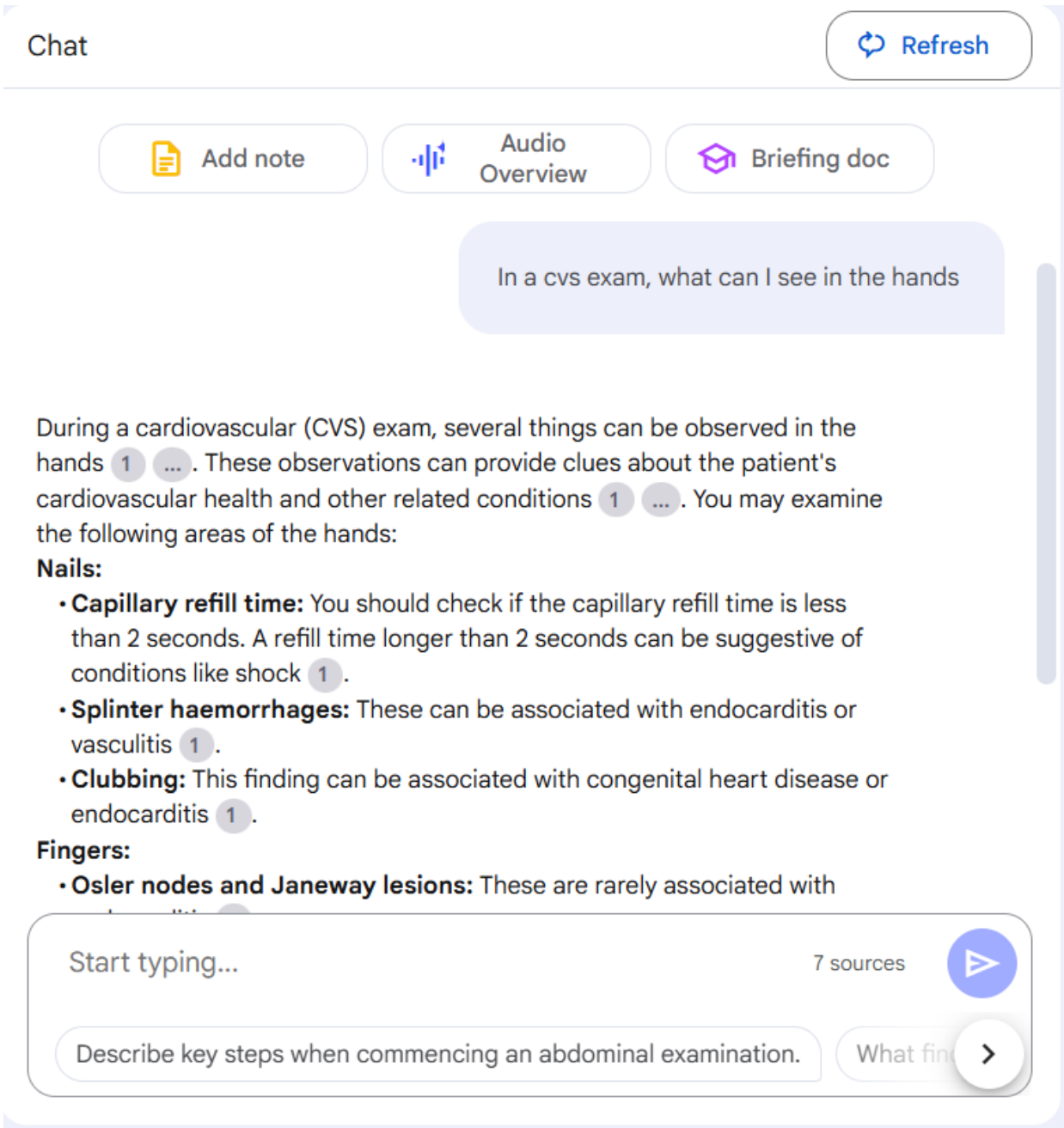

The model will try to answer your question based off the uploaded examination documents. Clicking on the citations will show you where in the uploaded documents NotebookLM got its information from.

5) Have a play around using NotebookLM to help you learn the knowledge behind clinical examinations. We'll invite you to let us know how you got on after your practical exam. There are some example uses below.

Example uses

1) Revising a single examination

You can use NotebookLM to focus on a single examination, for example:

- By asking about procedure e.g. “In a CVS exam, what do I examine after the hands?”
- By asking about signs, symptoms or Ix e.g. “In a CVS exam, what do I look for in the legs?” or “what further investigations could I suggest?”

> In a CVS exam, what signs do I look for in the legs

During a cardiovascular (CVS) exam, when examining the **legs (lower peripheries)**, you should look for the following signs [1]:

- **Pitting oedema**: This can be suggestive of fluid overload [1]. You should note the **upper extent of the oedema** [1].
- **Saphenous vein harvesting**: Evidence of this, such as scars, suggests the patient has had a **Coronary Artery Bypass Graft (CABG)** [1].

2) Revising multiple examinations

NotebookLM is good at synthesising and comparing information across different exams, for example try:

- Asking it to identify aspects across exams “in which exams do I check for lymph nodes?”
- Asking it to contrast different exams “what’s the difference between what I look for in the hands in a CVS and resp exam?” or “Generate a table comparing reflexes tested in UL and LL exam”

Upper Limb Reflex	Lower Limb Reflex
**Biceps reflex** [1]	**Knee jerk** [2]
**Triceps reflex** [3]	**Ankle jerk** [4]
**Supinator reflex** [3]	**Plantar reflex (Babinski response)** [4]
Wrist clonus (assessed if brisk reflexes) [5]	Ankle clonus (assessed if reflexes are abnormally brisk) [6]

The table above compares the reflexes explicitly tested using a tendon hammer in the upper limb peripheral neurological examination [1] [...] and the lower limb peripheral neurological examination [2] [...] as described in the sources. Both examinations also mention clonus (wrist in UL [5] and ankle in LL [6]) as a sign of an upper motor neuron lesion, assessed by briskly moving the joint [5] [...].

3) Testing your knowledge

NotebookLM can be used as a tutor to generate questions or quiz you, for example:

"Generate some short questions with answers, testing the neuro exams"

Here are the short questions testing the neurological exams, drawing on information from the sources:

- During the general inspection of the lower limb neuro exam, what does **cachexia** potentially indicate?
- In the upper limb neuro exam, what might **cogwheeling** at the wrist suggest?
- According to the MRC scale, what does a power grade of **2** indicate?
- When testing the **ankle jerk reflex**, how should the patient be positioned?
- What is being tested when a patient is asked to walk **heel to toe (tandem gait)**?
- When assessing **vibration sensation**, where should you start testing in the lower limb?
- What is the significance of an **extensor response** during the plantar reflex test?
- How do you test for **elbow flexion** and what is the primary nerve root involved?
- What is **dysdiadochokinesia** testing for?
- What might **foot drop** during gait assessment indicate?

If you'd like MCQs in the proper format, ask NotebookLM to refer to the style guide included:

"Using the style guide, generate 5 MCQs across all exams"

Here are 5 MCQs across all the provided exam protocols, following the style guide and formatted for better readability:

- **A 60-year-old patient with a history of chronic liver disease is noted to have a flapping tremor of the wrists when asked to extend their arms.**
- **Which of the following signs is also commonly associated with chronic liver disease?**
- A. Asterixis B. Carotid bruit C. Koilonychia D. Palmar pallor E. Splinter haemorrhages

----

- **A 72-year-old man is being examined for a cardiac complaint. On inspection of his hands, the capillary refill time is noted to be greater than 2 seconds.**
- **This finding is suggestive of which of the following?**
- A. Hyperlipidaemia B. Normal perfusion C. Peripheral cyanosis D. Poor perfusion (e.g., shock) E. Vasculitis

These are just some examples of how to use NotebookLM. Please feel free to experiment with the tool, we'd be interested to hear how you used it.

FAQ

1. What is NotebookLM?

NotebookLM is a large-language model (LLM) tool created by Google, based on its Gemini model. It uses retrieval-augmented generation, meaning it answers questions specifically based on documents you upload.

2. How can NotebookLM help me revise clinical examinations?

You can ask NotebookLM specific questions about clinical examinations, compare different examinations, or request practice questions and quizzes based on the uploaded documents.

3. Is NotebookLM free to use?

Yes, NotebookLM is completely free to use; all you need is a Google account.

4. What documents should I upload?

You should upload the provided six examination protocol PDFs and the MCQ style guide available in the 'examinations for upload folder'.

5. How accurate is NotebookLM?

NotebookLM's accuracy is improved by limiting responses to the content of uploaded documents. However, you should always verify information using course material and textbooks.

6. Should I input personal data into NotebookLM?

No. It is strongly recommended that you do not input personal data (e.g., names, dates of birth, addresses) into NotebookLM.

7. How do I know where NotebookLM's answers come from?

NotebookLM provides citations linked directly to the source documents you uploaded. Clicking these citations will show the relevant sections.

8. Can I use NotebookLM to generate multiple-choice questions (MCQs)?

Yes. Using the provided MCQ style guide document, you can ask NotebookLM to generate properly formatted MCQs.

9. Where can I access NotebookLM?

Visit https://notebooklm.google.com to get started.

10. I get a 'service unavailable' message when trying to use NotebookLM, how do I fix this?

The most likely fix for this is to verify your age on your Google account

11. Do I have to participate in the research study to use NotebookLM

No, participation in the study is entirely voluntary, but we would love to hear how you got on using NotebookLM. We will advertise focus groups and surveys in late April/May.

12. Who can I contact if I have more questions?

If you have further questions, please contact rohin.athavale@bristol.ac.uk.
