# Supplementary material for: Medical Students’ Perceptions of Retrieval-Augmented Generation and Large Language Models: Dyadic Interview and Exploratory Survey Study
Source: JMIR Form Res. 2026 Oct 30;10:e106157. doi: 10.2196/106157 (PMC13722098; doi:10.2196/106157)
Supplement: Supplementary file 2 [file formative-v10-e106157-s002.docx]

Survey questions

**Introduction:**

Thank you for participating in this survey. Your feedback will help us understand how large language models can help support medical students.

This survey should take about 5-10 minutes to complete.

**Section 1: Consent form**

**Section 2: General AI Views**

1. **Excluding NotebookLM, how often do you use AI tools (e.g., ChatGPT) in your studies?**
   - Daily
   - Multiple times a week
   - A few times a month
   - Rarely
   - Never
2. **Excluding NotebookLM, how useful do you find AI tools for your learning?**
   - Very useful
   - Somewhat useful
   - Neutral
   - Somewhat not useful
   - Not useful at all
3. **Please share any general thoughts or concerns you have about using AI in education.**
   - [Open text box]

**Section 3: Experience with NotebookLM**

4. **How frequently did you use NotebookLM while it was available?**
   - Daily
   - Multiple times a week
   - A few times a month
   - Rarely
   - Never
5. **How useful did you find NotebookLM?**
   - Very useful
   - Somewhat useful
   - Neutral
   - Somewhat not useful
   - Not useful at all
6. **Which features of NotebookLM did you find most useful? (Select all that apply)**
   - Summarising information
   - Answering questions
   - Generating study materials
   - Connecting related concepts
   - Other: [Open text box]
7. **Please describe any specific ways in which NotebookLM helped you with your studies.**
   - [Open text box]
8. **How reliable did you find the information provided by NotebookLM?**
   - Very reliable
   - Somewhat reliable
   - Neutral
   - Somewhat unreliable
   - Very unreliable
9. **Did you encounter any challenges or issues while using NotebookLM?**
   - [Open text box]

**Section 4: Comparison with traditional study methods**

By traditional study methods, we mean any non-AI based methods of learning, including but not limited to lectures, textbooks, flashcards etc.

10. **How does using NotebookLM compare to traditional study methods in terms of effectiveness?** Where effectiveness refers to the ability to facilitate the learning and retaining of knowledge
    - Much less effective
    - Somewhat less effective
    - About the same
    - Somewhat more effective
    - Much more effective
11. **How does using NotebookLM compare to traditional study methods in terms of efficiency**? Where efficiency refers to the quantity of material you are able to cover in a given time
    - Much less efficient
    - Somewhat less efficient
    - About the same
    - Somewhat more efficient
    - Much more efficient
12. **If you had access to a tool such as NotebookLM, would you favour it over traditional study methods?**
    - Yes
    - No
    - Unsure

**Section 5: Ethical and Practical Considerations**

13. **How concerned are you about your data when using AI tools like NotebookLM for your studies?**
    - Very concerned
    - Somewhat concerned
    - Neutral
    - Somewhat unconcerned
    - Very unconcerned
14. **How concerned are you about the environmental impact of using AI tools like NotebookLM for your studies?**
    - Very concerned
    - Somewhat concerned
    - Neutral
    - Somewhat unconcerned
    - Very unconcerned
15. **Please elaborate on any ethical concerns you have regarding the use of AI in medical education.**
    - [Open text box]
16. **Are there any improvements or additional features would you suggest for a tool such as NotebookLM to better support your learning?**
    - [Open text box]
17. **Would you recommend using NotebookLM to other medical students?**
    - Yes
    - No
    - Unsure

**Conclusion:** Thank you for your time and valuable feedback. Your responses will help us improve the use of AI tools in medical education.

If you would like to be entered into the prize draw for £20 vouchers, please enter your email address below: [ ]
