# Supplementary material for: Medical Students’ Perceptions of Retrieval-Augmented Generation and Large Language Models: Dyadic Interview and Exploratory Survey Study
Source: JMIR Form Res. 2026 Oct 30;10:e106157. doi: 10.2196/106157 (PMC13722098; doi:10.2196/106157)
Supplement: Supplementary file 3 [file formative-v10-e106157-s003.docx]

### **Topic guide**

| **Topic** | | **Prompts** |
| --- | --- | --- |
| Introduction | Welcome | *Welcome*  *Explain structure and topics*  *Ground rules & confirm consent* |
|  | Icebreaker | Can you briefly introduce yourself and state if you have ever used any AI tools or applications in your studies? If so, which ones? |
| Section 1: General views and use of AI and LLMs | Views on AI | What are your general thoughts on AI?  How do you feel about the increasing use of AI in education and healthcare?  What potential benefits and challenges do you see with AI in general? |
|  | Views on LLMs | Are you familiar with large-language models (LLMs) like ChatGPT? If so, how have you used them?  What do you think are the strengths and weaknesses of LLMs in the context of learning and education?  How do you perceive the accuracy and reliability of information generated by LLMs? |
|  | Aspirations on how LLMs could be used to help studies | How do/would you use LLMs to help you study?  What would you like LLMs to do to help you further? |
| Section 2: Experience with NotebookLM | General experience | Can you describe your experience using NotebookLM for learning clinical examinations?  What specific features of NotebookLM did you find most useful or beneficial?  Were there any features that you found less useful or challenging to use? |
|  | Perceptions of accuracy and reliability | How accurate and reliable did you find the information generated by NotebookLM?  Were there any instances where you questioned the accuracy of the content provided by NotebookLM? |
|  | Ethics | How do you feel about your data being submitted to a LMM? |
|  | Comparison to traditional methods | How does using NotebookLM compare to traditional study methods you have used in the past?  Did you find NotebookLM to be more efficient or effective in any particular areas? |
|  | Impact on learning | Do you feel that using NotebookLM has improved your understanding and retention of clinical examination material?  How has NotebookLM influenced your confidence and preparedness for clinical exams?  What other areas would you use NotebookLM for in your learning? |
|  | Suggestions for improvements | What improvements or additional features would you suggest for NotebookLM to better support your learning? |
| Closing |  | Is there anything else you would like to share about your experience with NotebookLM?  *Chance for questions.*  *Thank participants for their time and input.* |
